# The COVID-19 pandemic, food insecurity, and psychosocial well-being in young South Africans newly diagnosed with HIV: a mediation analysis

**DOI:** 10.21203/rs.3.rs-4560188/v1

**Published:** 2024-06-27

**Authors:** Connor Bondarchuk, Tiffany Lemon, Andrew Medina-Marino, Elzette Rousseau, Siyaxolisa Sindelo, Nkosiypha Sibanda, Lisa Butler, Linda-Gail Bekker, Valerie Earnshaw, Ingrid Katz

**Affiliations:** Harvard University; Arizona State University; Desmond Tutu HIV Foundation; Desmond Tutu HIV Foundation; Desmond Tutu HIV Foundation; Desmond Tutu HIV Foundation; Queen’s University; Desmond Tutu HIV Foundation; University of Delaware; Harvard University

**Keywords:** food insecurity, HIV/AIDS, AYAs, depression, anxiety, self-esteem, socioeconomic factors, COVID-19

## Abstract

**BACKGROUND:**

Poor psychological well-being, including depression, anxiety, and low self-esteem, is both prevalent among young South Africans living with HIV and associated with poor HIV clinical outcomes. By impacting food insecurity and employment, the COVID-19 pandemic may have influenced psychological well-being in this population. This analysis sought to examine whether food insecurity and unemployment mediated the relationship between study cohort (pre- versus during-pandemic) and psychological well-being in our sample of young South Africans living with HIV.

**METHODS:**

This was a secondary analysis comparing baseline data from two cohorts of young South Africans ages 18–24 from the Cape Town and East London metro areas who tested positive for HIV at clinics (or mobile clinics) either before or during the COVID-19 pandemic. Baseline sociodemographic, economic, and psychological outcomes were analyzed through a series of bivariate logistic regression and mediation analyses. All data were analyzed in 2023 and 2024.

**RESULTS:**

Reported food anxiety, insufficient food quality, and insufficient food quantity were lower in the cohort recruited during the COVID-19 pandemic than those recruited before the pandemic (p<0.001). Higher levels of food insecurity predicted higher depressive and anxiety symptoms and lower self-esteem. Food anxiety, insufficient food quality, and insufficient food quality, but not unemployment, mediated the relationship between study cohort and depressive symptoms, anxiety symptoms, and self-esteem.

**CONCLUSION:**

Food insecurity may have decreased amongst our sample of young people during the COVID-19 pandemic. Our findings build on our understanding of how the psychological well-being of young people living with HIV was affected by the COVID-19 pandemic and may lend support to interventions targeting food insecurity to improve psychological well-being in this population.

## INTRODUCTION

With more than 150,000 new infections per year^1^, South Africa is home to the world’s largest population of people living with HIV (PLWH)^2^. Compared to other age cohorts, adolescents and young adults (AYA) experience worse HIV clinical outcomes, including high mortality rates^3^ and lower rates of antiretroviral therapy initiation^4^, antiretroviral adherence^5,6^ and viral suppression^4,6^. In addition to navigating an inherent set of challenges related to the transition from childhood into adulthood (i.e. becoming self-sufficient, engaging in identity formation)^7–9^, AYA living with HIV face disproportionate socioeconomic (lack of economic independence, difficulty accessing clinics), and psychosocial (difficulty adapting to sustained medication, higher rates of stigma), barriers that may complicate HIV care initiation and antiretroviral therapy (ART) adherence^5,10^. These barriers may not only contribute to poor HIV clinical outcomes but may also manifest in the high rates of poor psychological well-being observed among AYA living with HIV^11–16^.

A particularly acute barrier recently faced by AYA was national lockdowns due to COVID-19, which may have further contributed to poor health outcomes in this population. Indeed, the psychological well-being of AYA living with HIV may have been further impacted by disruptions related to the COVID-19 pandemic and subsequent infection control measures^17–18^, which disrupted access to mental health care, social support networks, and employment opportunities^19–23^. These effects may have been particularly pronounced in South Africa, which in March 2020 instituted a stringent lockdown involving the banning of nonessential travel outside of the home, the closure of schools and businesses, and a prohibition on all public gatherings^24,25^. The initial five-week lockdown was followed by the implementation of a staged system of social and economic restrictions that ended only in April 2022 and whose stringency depended on provincial rates of COVID-19 infection. The economic toll of the lockdown measures included the loss of an estimated 3 million jobs^26^, predominantly affecting South Africans from key and vulnerable populations, including PLWH^27–29^.

Social stress theory (SST) provides a useful framework for understanding how macrolevel economic processes occurring during the pandemic may have influenced stress processes at the individual level, thus promoting differential levels of psychosocial well-being across social groups^30^. According to the social stress framework, economic disruptions, such as those experienced during the COVID-19 pandemic, may lead to individually experienced life-events (unemployment, food insecurity) that in turn may promote psychological stress. However, social stress theory also holds that the degree to which these economic disruptions promote poor psychological and social functioning depends greatly on the distribution of social vulnerabilities; that is, the relationship between economic disruption and psychological distress is moderated greatly by one’s health, sociodemographic, and other personal characteristics (e.g., physical health, gender, and ability to cope). Taken in the context of our study population of young South African adults recently diagnosed with HIV, social stress theory thus suggests that AYA with HIV may be at particular risk of poor psychosocial outcomes related to COVID-19-induced economic disturbances.

To our knowledge, there have been no studies evaluating the economic vulnerability of AYA living with HIV during the COVID-19 pandemic. While several studies have elucidated the effect of social distancing and isolation on depression and anxiety^31–33^, few have evaluated the degree to which COVID-19-related economic disruptions affected psychological and social functioning in youth living with HIV. Considering that depression^34^, anxiety^35^, and self-esteem^36^ have been found to be directly associated with worse HIV clinical outcomes, it is critical to understand the broader structural forces affecting psychological and social functioning in AYA living with HIV during the COVID-19 pandemic.

To determine the relationships among the COVID-19 pandemic, economic vulnerability, and psychological well-being of AYA living with HIV, this secondary analysis sought to (1) compare levels of food insecurity and unemployment between a pre- and intra-pandemic cohort of AYA living with HIV; (2) determine whether key baseline demographic factors are predictors of economic vulnerability; and (3) determine whether food insecurity and unemployment mediated the relationship between time of diagnosis in relation to the COVID-19 pandemic and psychological well-being. In particular, we hypothesized that relative to AYA diagnosed with HIV before the pandemic, those diagnosed during the pandemic would exhibit higher levels of food insecurity and unemployment. In addition, based on SST’s premise that macrolevel economic disruptions can provoke psychological distress, we hypothesized that food insecurity and unemployment would significantly mediate the relationship between time of diagnosis and psychological well-being in this population.

## METHODS

### Population

The data for this secondary analysis come from a sequential prospective cohort study and a subsequent pilot randomized controlled trial, which was conducted as part of a larger study called Standing Tall (NIH 1R34MH114897–01A1). The first study was conducted in Cape Town in 2018–2019, and the second study was conducted in Cape Town and East London, South Africa in 2020–2022. Both studies recruited 100 newly diagnosed HIV positive participants aged 18–24 years. The data for these analyses were drawn from baseline surveys, which were conducted at the time of enrollment in both studies.

Participants in the first study were recruited from community-based centers and well-established mobile clinics operating in and around several communities in Cape Town. The mobile clinics offered a variety of services including family planning, HIV testing and counseling, and STI testing and treatment. Participants in the second study were recruited from the same mobile clinics operating in and around Cape Town, in addition to three clinics in the East London area.

To be eligible for participation in the studies, individuals had to be ART naïve, speak English and/or isiXhosa, and reside in the Cape Town or East London (for the second study) metro areas. Individuals who were currently pregnant or diagnosed with TB disease, and persons under the age of 18 years were excluded. Individuals in the pre-COVID cohort were recruited between 2018 and 2019 and those in the during-COVID cohort were recruited between 2020 and 2022.

### Measures

#### Primary Predictors

Time of diagnosis in relation to the pandemic was the primary predictor in most of our analyses. Based on the trial in which they participated, study participants were categorized into dichotomous pre- and during-COVID groups

Additional dichotomous baseline characteristics included: age (≥21 years vs. <21 years), gender (male vs. female), education level (did not complete high school vs. completed high school) and living status (alone vs. with others) were used as dichotomous predictor variables.

#### Psychosocial Outcomes

Three psychosocial measures were assessed in this analysis.

1. *Depressive symptoms*. The 9-question Patient Health Questionnaire (PHQ-9) was utilized to measure the severity of depressive symptoms^37^. Only baseline measures were used in this study. The 9 items assess symptoms associated with the diagnosis of clinical depression. Responses on each item range from 0 (‘not at all’) to 3 (‘nearly every day’). Higher scores reflect greater degrees of depressive symptoms, with scores ≥ 10 indicating moderate-severe depressive symptoms. In this analysis, we used the continuous PHQ-9 total score. Cronbach’s alpha for the PHQ-9 questionnaire was α = 0.920.
2. *Generalized anxiety symptoms*. The 7-item Generalized Anxiety Disorder (GAD-7) Scale was used to identify symptoms of generalized anxiety disorder (GAD) and determine the severity of symptoms known to be associated with GAD^38^. Responses to each item range from 0 (‘not at all’) to 3 (‘nearly every day’), with total scores ranging from 0 to 21. Higher scores reflect a greater degree of anxiety symptoms, with scores ≥10 reflecting moderate-severe anxiety symptoms. In our analyses, continuous GAD-7 total scores were used. Cronbach’s alpha for the GAD-7 questionnaire was α =0.886.
3. *Self-esteem*. The Rosenberg Self-Esteem Scale (RSES) was used to evaluate individual self-esteem^39^. All items are answered using a 4-point Likert scale with scores ranging from 1 (‘strongly disagree’) to 4 (‘strongly agree’). Five of the items, reflecting negative feelings about the self, are reverse scored and totaled with the other items to create a composite score. Total RSES scores were utilized as a continuous outcome variable throughout the study. Lower scores indicate higher degrees of self-esteem/self-efficacy. Cronbach’s alpha for the RSES questionnaire was α = 0.724.

#### Economic Variables

Two measures of economic well-being were assessed at baseline.

1. *Food insecurity* was divided into three separate domains, including food anxiety, insufficient quality, and insufficient quantity. Each of the items representing these three domains were taken from the Household Food Insecurity Access Scale (HFIAS), which asks respondents to recall whether they have experienced different aspects of food insecurity, including feelings of anxiety over food and perceptions that food is of insufficient quantity and quality (including diversity or nutritional preference), within a recall period of 30 days (4 weeks)^40^. All items are answered using a 4-point Likert scale format with answers ranging from 1 (‘never,’ i.e., 0 times a month) to 4 (‘often,’ i.e., 10x or more per month), with total scores ranging from 3 to 12. Answers to each domain were dichotomized to create a binary variable presenting high (‘often,’ ‘sometimes’) or low (‘rarely,’ ‘never’) levels of anxiety, insufficient quality, and insufficient intake. In our logistic regression analysis, total food insecurity was dichotomized into high food insecurity (scores ≥6, indicating that the participant experienced food insecurity more than 1–2x in the past month) and low food insecurity (scores ≤6, indicating that the participant experienced food insecurity on average less than 1–2x in the past month).
2. *Employment status* was treated as a categorical variable based on responses to the question ‘are you currently employed’?. In this analysis, employment was treated both as binned dichotomous variable (employed vs. unemployed).

#### Covariates

Our directed acyclic graph (DAG) analysis did not identify any covariates that could reasonably act as confounders of the relationship between time of diagnosis and our psychosocial outcomes of interest.

### Statistical analysis

Descriptive statistics were used to describe study participants’ baseline demographic and economic characteristics. For dichotomous characteristics, chi-square tests of association were conducted to determine whether a statistically significant association existed between time of diagnosis (pre- vs. during-COVID-19) and each characteristic. For continuous characteristics, a two-sided independent sample t-test was utilized to evaluate whether means were statistically different between the two cohorts.

Following aggregation of the two datasets, bivariate logistic regression analysis was performed in which four dichotomous socio-demographic predictor variables (eg., age, gender, living status, and education level) were used to predict dichotomous levels of food insecurity (high/low) and unemployment (yes/no). Odds ratios were calculated to determine the constant effect of our predictors on the likelihood of an outcome (i.e. having food insecurity or unemployment). The Wald test was used to determine the statistical significance of each of the predictor variables.

**Figure 1:**
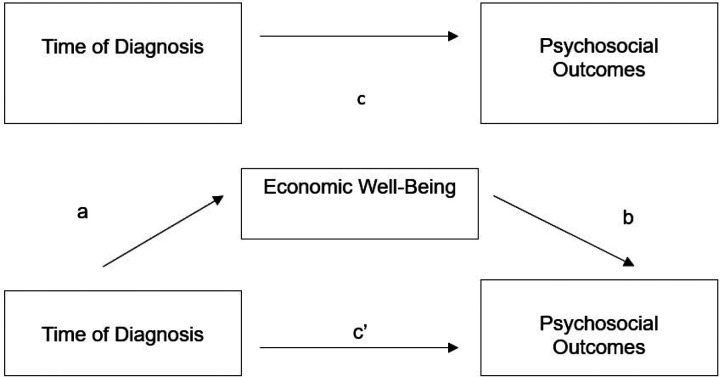
Diagram of proposed mediation model

Mediation analyses, informed by Baron and Kenny^41^, were conducted to test the mediation effect of each of our economic variables on the association between time of diagnosis and our four continuous psychosocial outcomes (see Figure 1). First, the direct effect of time of diagnosis on each psychosocial outcome was determined (path c). Next, we determined whether time of diagnosis significantly predicted our mediator of interest (path a). Then, we determined whether our mediator of interest predicted our psychosocial outcome (path b). Finally, we determined if time of diagnosis remained a significant predictor of each psychosocial outcome after controlling for our mediator of interest (path c’). Full mediation occurs when the association between predictor and outcome is no longer statistically significant, whereas partial mediation occurs if the strength of association is reduced but still significant. The Sobel test was used to determine whether the mediating effects of each economic variable was statistically significant^42^. All data were analyzed in the years 2023 and 2024.

## RESULTS

All 200 of our participants identified as Black African and identified English or isiXhosa as their preferred language. The median age of the pre- and during-COVID cohorts were 22 (IQR: 19–23) and 21 (IQR: 19–22), respectively. Mean ages were similar in each study cohort (21.17 years in the pre-COVID cohort vs. 20.68 years in the during-COVID cohort, p=0.078). Gender breakdown (c^2^=0.207, p>0.05), education level (c^2^=0.322, p=0.570), and living status (c^2^=3.269, p=0.071) was not significantly associated with time of diagnosis (Table 1).

In terms of economic characteristics of our two cohorts, there was no statistically significant association between employment status and time of diagnosis (c^2^=1.339, p=0.247). However, being diagnosed during the pandemic was significantly associated with less reported food anxiety (c^2^=78.95, p<0.001), less reported insufficient food quality (c^2^=46.07, p<0.001), and less insufficient food quantity (c^2^=52.35, p<0.001).

In bivariate regression analyses (Table 2), female participants had a 62.6% lower odds of reporting high food insecurity than male participants (OR 0.374, CI 0.156–0.899, p=0.028). Female gender (OR 2.778, CI 1.088–7.095, p=0.033) and living alone (OR 4.666, CI 1.793–12.138, p=0.002) were associated with significantly greater odds of unemployment whereas completing high school was associated with significantly lower odds of unemployment (OR 0.333, CI 0.142, 0.784, p=0.012).

Results from the mediation analyses evaluating the effect of our mediator on the relationship between our economic variables (food anxiety, insufficient food quality, and insufficient food quantity, and unemployment) and each of our three psychosocial outcomes are presented in Tables 3–5. Being diagnosed with HIV during the COVID-19 pandemic did not significantly predict depressive symptoms as measured by PHQ-9 scores (β=0.214, p=0.748) (Table 3) or anxiety symptoms as measured by GAD-7 scores (β=0.325, p=0.586) (Table 4). However, diagnosis during the COVID-19 pandemic did predict higher self-esteem, which was associated with lower RSES scores (β=−5.409, p=<0.001) (Table 5).

Partial mediation of the relationship between time of diagnosis and PHQ-9 score was observed when food anxiety (Sobel z = −3.87, p<0.001), insufficient food quality (Sobel z = −3.13, p=0.0017), and insufficient food quality (Sobel z = −2.12, p=0.034) were included in our regression model (Table 3). Similarly, food anxiety (z=−3.75, p<0.001), insufficient food quality (−3.16, p=0.0016), and insufficient food quantity (z=−2.61, p=0.0091) all exhibited a partial mediation effect on the relationship between time of diagnosis and GAD-7 scores (Table 4). In addition, partial mediation of the relationship between study cohort and self-esteem (RSES scores) were observed when food anxiety (Sobel z = −2.11, p=0.035), insufficient food quality (Sobel z = −2.40, p=0.017), and insufficient food quantity (Sobel z = −3.07, p=0.002) were included in our model (Table 5).

Unemployment did not exhibit a significant mediation effect on the relationship between time of diagnosis and any of our psychosocial variables.

## DISCUSSION

The major aims of this analysis were to compare the economic well-being of a pre- and during-COVID-19 cohort of newly diagnosed AYA living with HIV and to determine whether measures of economic well-being mediated the relationship between time of diagnosis and psychological well-being. Although it was hypothesized that diagnosis during the COVID-19 pandemic would be associated with poorer economic well-being, the results of our analyses of baseline food insecurity and unemployment demonstrated that diagnosis during the pandemic was associated with a significantly lower level of reported food anxiety, insufficient food quality, and insufficient food quantity. No association was found between time of diagnosis and unemployment.

Concordant with our predictions based on social stress theory, our mediation analyses revealed that higher levels of food insecurity significantly predicted higher levels of depressive symptoms, anxiety symptoms, and lower self-esteem. Although diagnosis during the pandemic predicted only higher self-esteem, lower levels of food insecurity were associated with better overall psychological functioning. On the other hand, unemployment was not a significant predictor of any of our psychosocial outcomes. Interestingly, our results thus suggest that food security may have a more profound impact on psychosocial outcomes than employment status in this population. On the whole, while our findings do not indicate a relationship between unemployment and poor psychological well-being in this population, they do lend support to a previously identified relationship between food insecurity and indicators of poor psychological well-being known to be related to HIV treatment outcomes^43–44^.

Our results also add to our understanding of the understudied and likely complex relationships among the pandemic, macrolevel economic disruptions, and psychological outcomes among newly diagnosed young South Africans living with HIV. First, they suggest that at least among this population of urban youth newly diagnosed with HIV, reported food anxiety and insufficient food quality and quantity decreased during the COVID-19 pandemic, despite a background of increased unemployment^45,46^ and food insecurity in South Africa overall^28, 45, 47^. Though surprising, data collected by the South African government during the pre- and during-COVID-19 time periods does suggest some degree of heterogeneity in the effects of the pandemic on food insecurity, with some provinces experiencing greater food insecurity and others demonstrating declines during the pandemic^48^.

Conceivably this lower food insecurity observed in our intra-pandemic cohort may be explained by the disproportionate insulating effect of pandemic-era government transfer payments on the income of poor, unemployed South Africans^46^, who composed the majority of both of our cohorts. Indeed, in April 2020, South Africa’s government instituted both a series of existing grant top-ups in addition to a new Social Relief of Distress Grant of R350 ($21), which was offered to South Africans aged 18 and above who were already unemployed or who had lost employment due to the country’s lockdown^49^. Given that over 8 million economically vulnerable South Africans benefited from this 41 billion rand ($2.4 billion USD) social assistance package^50^, it is plausible that the receipt of these grants had a particularly protective effect on our economically disadvantaged population of AYA. This increase in government support may have been complemented by both the efforts of local nongovernmental organizations, which engaged in extensive community outreach to alleviate intrapandemic food insecurity through the distribution of food and food vouchers^51–52^, and a notable increase in household agricultural production during the COVID-19 pandemic^48^. Thus, while our results may point to the well-documented efficacy of South Africa’s robust system of unconditional cash transfer payments^45, 53–56^ on hunger^57^, nongovernmental and individual efforts may have also contributed to the decline observed within this population.

Our findings suggest that the decrease in food insecurity, whether through an expanded social safety net, nongovernmental support, or other means, may have had a protective effect on mental health outcomes during the COVID-19 pandemic. In doing so, our results not only align with a growing body of research suggesting an association between food insecurity and poor mental health outcomes^58–60^, but also indicate that improvements in food security may have buffered against the psychological distress associated with the COVID-19 pandemic^61–62^. Put another way, whereas social stress theory predicts that limited economic resources may lead to increased distress and poor psychological health^30^, our results suggest that the reverse may also be true: that lower levels of food insecurity may facilitate improved psychological functioning despite the presence of a globally significant macrolevel stressor. Interestingly, the mediating effect of declining food insecurity on psychosocial well-being was strongest for self-esteem, a psychological outcome for which an association with food insecurity has not been clearly established. While poverty in general has been shown to be negatively associated with self-esteem^63–64^, further research is needed to identify the mechanism by which food security may affect one’s perception and confidence in oneself.

Overall, our results may lend further support for structural interventions, policies, and programs that improve access to quality food among people living with HIV. Although the impact of undernutrition and poor HIV clinical outcomes has been well-established^65–67^, our results as well as those of a growing body of literature suggest that the benefits of improved food security may also extend to key indicators of mental health^68–71^. Moreover, though there remains a dearth of best practice studies evaluating specific intervention strategies to address the HIV-food insecurity syndemic^72^, initiatives that provide direct monetary or food assistance have demonstrated particular efficacy in improving food security in the sub-Saharan African context^73–75^. Thus, given the association of food insecurity with both poor physical and mental health outcomes in PLWH, such direct provision interventions may be especially potent in terms of their ability to improve multiple, overlapping domains of health.

### Strengths, limitations, and future directions

This study has several key strengths. For one, our study is one of the first to investigate changes in psychosocial and economic well-being across the pandemic among AYA living with HIV. Indeed, the timing of our cross-sectional surveys, which were conducted among two demographically similar South African samples of AYA before and during the pandemic, allowed us to evaluate important trends in economic and psychosocial well-being across this important juncture in time. In this manner, our analyses focusing on AYA living with HIV permitted the identification of intrapandemic changes that may have been particular to this unique population, and that may not have been observed in samples taken from the general population of PLWH. Our study thus provides important insight into the ways in which the pandemic may have uniquely impacted psychological and economic correlates of well-being among this vulnerable, disproportionately HIV affected population.

Nevertheless, despite identifying a promising association between food security and mental health amongst AYA living with HIV, our study has several limitations. First, given our focus on young people from urban South Africa, our results may not be generalizable to all South African PLWH. In fact, our own results suggest that levels of food insecurity may have differed significantly among different sub-populations of AYA living with HIV, such as young HIV positive men. Moreover, our mediation analyses were conducted using data from two cross-sectional surveys, and therefore, the directionality of our proposed mediator to outcome pathway cannot be directly assessed. Additionally, the time of diagnosis was treated as a dichotomous variable based on the recruitment of our two cohorts, and our classification of groups into pre- and during-pandemic cohorts may have disguised trends in food insecurity and or unemployment that may have changed longitudinally throughout South Africa’s pandemic-era State of Disaster. Finally, while our cohorts were recruited using similar procedures and did not differ significantly in terms of key sociodemographic characteristics (gender, unemployment status, education level, etc.), it is possible that the during-COVID cohort may have differed on the basis of other unmeasured factors. Therefore, we cannot rule out the possibility it may be that these unique characteristics of the group recruited during the pandemic, rather than the pandemic itself, that explain the lower food insecurity observed.

While our study adds to the literature about recent trends in food insecurity in South Africa, additional retrospective analyses of the effects of pandemic-era policies are needed to determine to what degree, if any, mental health and HIV clinical outcomes were affected by the increased economic support provided to vulnerable South Africans during the country’s COVID-19 lockdown. Public policy analyses investigating the differential effects of South Africa’s COVID-19 social grant system on subpopulations of South African PLWH would also be helpful in elucidating why AYA living with HIV may have been particularly buffered from the economic effects of the COVID-19 pandemic in comparison with other populations. Finally, translational research is needed to better understand how interventions targeting food insecurity may impact psychological and HIV clinical outcomes among PLWH.

## CONCLUSION

Our results demonstrated that in our sample of newly diagnosed South African AYA living with HIV, time of diagnosis during the COVID-19 pandemic was significantly associated with lower levels of food anxiety and insufficient food quality and quantity. Moreover, the relationships between time of diagnosis and psychological outcomes such as depressive symptoms, anxiety symptoms, and self-esteem were significantly mediated by food insecurity. Our findings build on our understanding of how the psychological well-being of AYA living with HIV was affected by the COVID-19 pandemic and lend support to interventions targeting food insecurity as a means of improving psychological outcomes in this vulnerable population.

## Figures and Tables

**Table 1.** Demographic and economic characteristics of young adults newly diagnosed with HIV by time of diagnosis (pre- vs. during-COVID).

Characteristics	Pre-COVID cohort	During-COVID cohort	c^2^ value (p-value)
**Total**	100	100	
**Age**			
Median age (years, IQR):	22 (19–23)	21 (19–22)	
Mean age (years, SD):	21.17 +/− 1.949	20.68 +/− 1.959	P=0.078
**Gender**			
Male	10 (10%)	16 (16%)	c^2^=0.207,
Female	90 (90%)	84 (84%)	P=0.207
**Education Level**			
Did not complete high school	44 (44%)	48 (48%)	c^2^=0.322
Completed high school	56 (56%)	52 (52%)	P=0.973
**Living Status**			
Living alone	15 (15%)	7 (7%)	c^2^=3.269,
Living with others	85 (85%)	93 (93%)	P=0.071
**Employment Status**			
Unemployed	81 (81%)	87 (87%)	c^2^=1.339,
Employed	19 (19%)	13 (13%)	P=0.246
**Food Insecurity**			
Reporting food anxiety			
Yes	**79 (79%)**	**35 (35%)**	**c** ^**2**^ **=78.95,**
No	**21 (21%)**	**65 (65%)**	**P<0.001**
Reporting insufficient quality			
Yes	**74 (74%)**	**30 (30%)**	**c** ^**2**^ **=46.07,**
No	**26 (26%)**	**70 (70%)**	**P<0.001**
Reporting insufficient quantity			
Yes	**74 (74%)**	**28 (28%)**	**c** ^**2**^ **=52.35,**
No	**26 (26%)**	**72 (72%)**	**P<0.001**

Boldface indicates statistical significance

**Table 2:** Logistic Regression Models of Relationship between Demographic Variables and Economic Variables (n=200)

Variable	OR	95% CI	P value
**Food Insecurity**			
Age <21	Reference	--	--
Age ≥ 21	0.752	(0.378, 1.496)	P=0.417
**Male**	**Reference**	--	--
**Female gender**	**0.374**	**(0.156, 0.899)**	**P=0.028**
Living with others	Reference	--	--
Living alone	0.923	(0.320, 2.663)	P=0.882
Did not complete high school	Reference	--	--
Completed high school	0.268	(0.346, 1.342)	P=0.268
**Unemployment**			
Age <21	Reference	--	--
Age ≥21	0.583	(0.272, 1.250)	P=0.166
**Male**	**Reference**	--	--
**Female gender**	**2.778**	**(1.088, 7.095)**	**P=0.033**
**Living with others**	**Reference**	--	--
**Living alone**	**4.666**	**(1.793, 12.138)**	**P=0.002**
**Did not complete high school**	**Reference**	--	--
**Completed High School**	**0.333**	**(0.142, 0.784)**	**P=0.012**

Boldface indicates statistical significance

**Table 3:** Simple Mediation Analysis for Relationship between Time of Diagnosis and PHQ-9 Scores

Mediator	b for path a (SE)	b for path b (SE)	b for total effect c (SE)	b for direct c’ (SE)	p-value of indirect effect
Food Insecurity: Anxiety	**−0.44 (0.063)** **	**2.960 (0.636)** ***	0.214 (0.664)	**1.889 (0.692)** **	**P<0.001**
Food Insecurity: Insufficient Quality	**−0.44 (0.064)** ***	**2.268 (0.644)** ***	0.214 (0.664)	**1.504 (0.711)** *	**P=0.0017**
Food Insecurity: Insufficient Quantity	**−0.46 (0.063)** ***	**1.450 (0.656)** *	0.214 (0.664)	1.117 (0.736)	**P=0.034**
Unemployment	−0.060 (0.052)	0.112 (0.905)	0.214 (0.664)	0.209 (0.667)	P=0.902

Boldface indicates statistical significance (*p<0.05, **p<0.01, ***p<0.001).

**Table 4:** Simple Mediation Analysis for Relationship between Time of Diagnosis and GAD-7 Scores

Mediator	b for path a (SE)	b for path b (SE)	b for total effect c (SE)	b for direct c’ (SE)	p-value of indirect effect
Food Insecurity: Anxiety	**−0.44 (0.063)** **	**2.559 (0.576)** ***	0.325 (0.597)	**1.809 (0.625)** **	**P<0.001**
Food Insecurity: Insufficient Quality	**−0.44 (0.064)** ***	**2.062 (0.580)** ***	0.325 (0.597)	**1.529 (0.638)** *	**P=0.0016**
Food Insecurity: Insufficient Quantity	**−0.46 (0.063)** ***	**1.637 (0.586)** **	0.325 (0.597)	**1.368 (0.655)** *	**P=0.0091**
Unemployment	−0.06 (0.052)	0.681 (0.814)	0.325 (0.597)	0.286 (0.600)	P=0.498

Boldface indicates statistical significance (*p<0.05, **p<0.01, ***p<0.001).

**Table 5:** Simple Mediation Analysis of Relationship Between Time of Diagnosis and RSES scores

Mediator	b for path a (SE)	b for path b (SE)	b for total effect c (SE)	b for direct c’ (SE)	p-value of indirect effect
Food Insecurity: Anxiety	**−0.44 (0.063)** ***	**1.217 (0.551)** *	**−5.409 (0.494)** ***	**−4.874 (0.546)** ***	**P=0.035**
Food Insecurity: Insufficient Quality	**−0.44 (0.064)** ***	**1.389 (0.543)** *	**−5.409 (0.494)** ***	**−4.798 (0.542)** ***	**P=0.017**
Food Insecurity: Insufficient Quantity	**−0.46 (0.063)** ***	**1.835 (0.542)** ***	**−5.409 (0.494)** ***	**−4.565 (0.542)** ***	**P=0.002**
Unemployment	−0.060 (0.052)	−0.456 (0.677)	**−5.409 (0.494)** ***	**−5.437 (0.46)** ***	P=0.581

Boldface indicates statistical significance (*p<0.05, **p<0.01, ***p<0.001).
